# Comparison of online marketing techniques on food and beverage companies’ websites in six countries

**DOI:** 10.1186/s12992-017-0303-z

**Published:** 2017-10-26

**Authors:** Marie A. Bragg, Margaret Eby, Josh Arshonsky, Alex Bragg, Gbenga Ogedegbe

**Affiliations:** 1 Department of Population Health, New York University School of Medicine, 227 East 30th Street Room 622, New York, NY 10016 USA; 2 New York University College of Global Public Health, 665 Broadway, 11th Floor, New York, NY 10003 USA

**Keywords:** Obesity, Food marketing, International

## Abstract

Food and beverage marketing contributes to poor dietary choices among adults and children. As consumers spend more time on the Internet, food and beverage companies have increased their online marketing efforts. Studies have shown food companies’ online promotions use a variety of marketing techniques to promote mostly energy-dense, nutrient-poor products, but no studies have compared the online marketing techniques and nutritional quality of products promoted on food companies’ international websites. For this descriptive study, we developed a qualitative codebook to catalogue the marketing themes used on 18 international corporate websites associated with the world’s three largest fast food and beverage companies (i.e. Coca-Cola, McDonald’s, Kentucky Fried Chicken). Nutritional quality of foods featured on those websites was evaluated based on quantitative Nutrient Profile Index scores and food category (e.g. fried, fresh). Beverages were sorted into categories based on added sugar content. We report descriptive statistics to compare the marketing techniques and nutritional quality of products featured on the company websites for the food and beverage company websites in two high-income countries (HICs), Germany and the United States, two upper-middle-income countries (UMICs), China and Mexico, and two lower-middle-income countries (LMICs), India and the Philippines. Of the 406 screenshots captured from company websites, 67·8% depicted a food or beverage product. HICs’ websites promoted diet food or beverage products/healthier alternatives (e.g. baked chicken sandwich) significantly more often on their pages (25%), compared to LMICs (14·5%). Coca-Cola featured diet products significantly more frequently on HIC websites compared to LMIC websites. Charities were featured more often on webpages in LMICs (15·4%) compared to UMICs (2·6%) and HICs (2·3%). This study demonstrates that companies showcase healthier products in wealthier countries and advertise their philanthropic activities in lower income countries, which is concerning given the negative effect of nutrition transition (double burden of overnutrition and undernutrition) on burden of non-communicable diseases and obesity in lower income countries.

**Electronic supplementary material:**

The online version of this article doi: (10.1186/s12992-017-0303-z) contains supplementary material, which is available to authorized users.

## Introduction

Poor dietary intake is associated with obesity [1]. Exposure to food and beverage marketing leads adults and children to increase short-term food consumption [2–4]. Although television food commercials continue to be a dominant source of exposure to food ads, food companies have increased their digital marketing presence as consumers spend more time online [5, 6]. Digital food marketing includes advertising through online advergames (e.g. website games intended to promote a brand), [7, 8] placement of ads on websites popular among specific demographic groups (e.g. children), [9–12] promotion of food products on branded websites, [13, 14] as well as through mobile devices and social media websites (e.g. Facebook) [15, 16]. These studies have shown that the majority of foods promoted on websites are energy-dense, nutrient-poor foods, [9–13, 16] and children who play online advergames are more likely to consume nutrient-poor snack foods and fewer fruits and vegetables, [7] and increase energy intake regardless of the health profile of the product being advertised [17].

Most digital food marketing studies have been conducted in the United States, despite increases in the promotion and sales of fast food and sugar sweetened beverages globally [18]. McDonald’s currently operates in more than 20,335 locations outside the United States, and it plans to open 3000 additional restaurants by 2020 with more than 1500 in China, Hong Kong, and South Korea [19, 20]. Similarly, Coca-Cola pledged to spend $5 billion in India on new plants and sales networks and a total of $17 billion in Africa by 2020 to increase its presence [21, 22]. Simultaneously, rates of obesity have risen in low- and middle-income countries, particularly among children and adolescents in developing countries, who have experienced an increased prevalence of overweight and obesity from 8·1 to 12·9% for boys and from 8·4 to 13·4% in girls [23]. One study showed a positive correlation between the number of Subway restaurants in a country and their national obesity rates [24]. Though that study is correlational and focused solely on lower-middle to higher income countries, the nutrition transition (i.e., moving from healthier, traditional and local foods, to more unhealthy Westernized diets) has led a number of health organizations to warn consumers of the damaging health effects associated with heavily processed energy-dense, nutrient-poor foods in contributing to the rise in global burden of obesity [25, 26]. Such warnings are strikingly similar to those resulting from the globalization of tobacco marketing, when tobacco companies began to market to developing countries in the 1970s after enforcement of stricter tobacco regulations in the U.S. [27].

Despite these warnings, there is little data on the variation in marketing strategies of food and beverage companies across high-income, low- and middle-income countries. In order to assess how these companies use marketing techniques across different countries, this study aimed to: (1) use a qualitative codebook to identify the types of marketing techniques used on 18 international fast food and beverage company websites; (2) examine the nutritional quality of the products marketed on those websites; and (3) use descriptive statistics to compare the marketing strategies and nutritional quality of promoted products across high-income countries (HICs), upper middle income countries (UMICs), and lower-middle income countries (LMICs).

## Methods

Six countries were chosen based on two criteria: 1) if McDonald’s, Coca-Cola, and KFC have been selling products in their country for more than 20 years; and 2) if the countries met criteria to be categorized as a high-, upper-middle-, or lower-middle-income country. These economic categories were based on the three classifications available through the Organization for Economic Co-Operation and Development (OECD): the United States and Germany (high income), China and Mexico (upper-middle-income), and India and the Philippines (lower-middle-income) [28]. Coca-Cola, McDonald’s, and Kentucky Fried Chicken (KFC) were chosen for analysis because they have the largest global market share, represent the fastest growing international brands, and began in the United States [29–33]. We included the latter criterion because of the role many U.S. food companies play in contributing to the nutrition transition through their global food marketing and promotion practices [34, 35].

In order to qualitatively analyze the marketing techniques used on different international food and beverage company websites, two researchers developed a qualitative codebook based on the content analysis guidelines described by Lombard and colleagues [36]. The codebook questions were based on similar qualitative food marketing studies [13, 14, 37, 38] and were designed to examine the following marketing techniques on the international company websites: visual depictions of food or beverage products, the use of charity references or logos, references to exercise or physical activity, child targeted images or themes (i.e. images of children, cartoon characters, or words synonymous with "child") , product promotions, or culturally relevant images or symbols. After the codebook was developed, 10 research assistants who did not participate in codebook development and were blind to the purpose of the study took screenshots in June 2016 of the 18 websites’ home pages and every page that could be accessed with one click from the website homepage. After compiling the screenshots, we trained four coders to view each screenshot and answer the codebook question based on the content shown in the screenshot. After training the coders, we randomly selected 10% of the total sample for the initial phase of coding that was needed in order to determine inter-coder reliability. An acceptable level of reliability was a Krippendorf’s alpha coefficient of 0·70 or higher, or inter-coder agreement levels of 90% or higher. Because all four coders rated the same random 10% of data points (i.e. screenshots from each webpage) from the total sample in order to establish reliability, four sets of potential codes were available for those 10% of screenshots [36]. Specifically, this means that each coder rated the same 41 screenshots to ensure the coders were interpreting the questions in the same way. The fact that these four coders coded the same 41 screenshots means that there were four sets of responses for those screenshots. Lombard and colleagues describe four approaches to handling discrepancies in this initial 10% of codes [36]. One approach involves randomly selecting codes from these four sets to make one final set that is representative of the group. We chose this method because any discrepancies between coders would likely reflect discrepancies in the interpretation of general consumers, but would not affect the reliability of ratings because the coders met the reliability criteria. Additionally, the “majority rules” only applies to scenarios with odd numbers of coders, so that approach was not used. And using an expert to “tie-breaker” or having a group discussion to change responses would mean that the natural variability that could likely reflects consumers’ variability in interpretations would be lost. Therefore, to ensure that a representative sample of these four sets of responses was integrated into the final data set, codes were randomly selected from these four sets. Thus, 10% of the final data set was made up of a random selection of codes from the reliability coding, and the remaining 90% of products were divided among four researchers and coded individually (Additional file 1: Figure S1). The dataset was analyzed using IBM Statistical Package for the Social Science (SPSS) version 23.0. We ran frequencies to determine the percentage of screenshots associated with each question in the qualitative codebook and chi-squared tests to determine whether statistically significant differences occurred between companies and countries. Fisher’s Exact Test was run when expected cell counts were too low for a chi-squared test.

In order to determine the healthfulness of each product endorsed on the websites, a nutrition score for each food product was generated from Nutrient Profile Model (NPM) [39, 40]. The NPM was selected because it was developed based on nutrition science and assigns a score to products based on nutrients to encourage (e.g. fiber) and nutrients to limit (e.g. sodium). It has been used in food marketing research focused on children and adults [38, 41, 42] and front-of-package food labeling studies [43–45]. Higher scores represent less healthful products, whereas lower scores represent products with healthier qualities. In order to translate the NPM score to an easy-to-understand scale, the final NPM score was converted to a Nutrient Profile Index (NPI), where *1* is the worst nutrition score and *100* is the best score. The NPI has been used in previous food marketing research [38, 41, 42]. The NPI uses the following formula: NPI score = −2 × NPM score + 70. A score ≥ 64 is considered healthier. One limitation of the NPM is that it codes many sugar-sweetened beverages (SSBs) similarly because sugar is the only ingredient. A sugar-sweetened beverage is defined as any beverage with “added sugar” (i.e. any caloric sweetener added to the beverage during the production process), and NPM gives SSBs similar scores even if the amount of added sugar differs. To provide more meaningful descriptions of beverage categories, we coded nonalcoholic beverages into 11 drink categories, three sugary drink subcategories, and five other drink categories as outlined in the Rudd Center’s Sugary Drink FACTS Report [46]. Drink categories included regular soda, fruit drinks, flavored water, sports drinks, iced tea, coffee, energy drinks, plain bottled water, 100% juice, diet drinks and light fruit juices. Drink subcategories included children’s drinks, full-calorie drinks, and reduced-sugar drinks. Because nutrition information was not available on every website, we also sorted the foods on all websites into the following broad and mutually exclusive categories: (1) non-diet food or beverage product (e.g. burger and fries), and (2) diet food or beverage product (e.g. salad, chicken sandwich). Then we categorized those same products into mutually exclusive subcategories to provide finer detail: (1) fried (i.e. any product that appeared breaded or fried); (2) cooked (i.e. any product that was baked, grilled, or broiled but not fried); (3) fresh (i.e. any raw product, such as produce, that was neither fried nor cooked). The latter categorization enabled us to make comparisons across all websites even in the absence of nutritional information. Food products were also coded and sorted into three mutually exclusive food categories: main dishes, side items, and garnishes (i.e. fresh fruits and vegetables, herbs and spices, or grains) in the image.

## Results

Across the 18 websites identified, we captured a total of 406 screenshots. Seven of the 18 websites displayed complete nutrition information for all products. Marketing techniques within brands varied between both individual countries and by the country’s economic groups, and some marketing techniques varied between brands (Table 1). Initially, the NPI model was used to score the nutritional quality of the foods, but because 11 of the websites lacked nutrition information, we supplemented this analytic approach by categorizing foods shown in the screenshots as possessing component/s that were: (1) fried (i.e. any product that appeared breaded or fried); (2) cooked (i.e. any product that was baked, grilled, or broiled but not fried); (3) fresh (i.e. any raw product, such as produce, that was neither fried nor cooked) (Table 2).

**Table 1:** Descriptive data on food and beverage company marketing techniques by economic classification and company

Company	Country (*N* = Total number of screenshots)	% of screenshots showing non-diet food or bev	% of screenshots showing diet food or beverage	% of screenshots link to a children section	% of screenshots containing a promotion	% of screenshots referencing exercise	% of screenshots referencing a charity
Coca-Cola	High Income (*N* = 17)	88.2 (*n* = 15)	41.2 (*n* = 7)	0 (*n* = 0)	29.4 (n = 5)	0 (*n* = 0)	0 (*n* = 0)
	Upper-Middle-Income (*N* = 24)	79.2 (*n* = 19)	29.2 (*n* = 7)	0 (*n* = 0)	0 (*n* = 0)*^a^	25 (*n* = 6)*^a^	4.2 (*n* = 1)
	Lower-Middle-Income (*N* = 48)	54.2 (*n* = 26)**	14.6 (*n* = 7)**^a^	0 (*n* = 0)	2.1 (*n* = 1)**^a^	6.3 (*n* = 3)	41.7 (*n* = 20)**
	Coca-Cola Total (*N* = 89)	67.4 (*n* = 60)	23.6 (*n* = 21)	0 (*n* = 0)	6.7 (*n* = 6)	10.1 (*n* = 9)	23.6 (*n* = 21)
McDonald’s	High Income (*N* = 27)	74.1 (*n* = 20)	40.7 (*n* = 11)	11.1 (*n* = 3)	22.2 (*n* = 6)	18.5 (*n* = 5)	7.4 (*n* = 2)
	Upper-Middle-Income (*N* = 86)	67.4 (*n* = 58)	11.6 (*n* = 10)*	65.1 (*n* = 56)*	18.6 (*n* = 16)	7.0 (*n* = 6)	3.5 (*n* = 3)
	Lower-Middle-Income (*N* = 86)	51.2(*n* = 44)**	7.0 (*n* = 6)**^a^	33.7 (*n* = 29)**	8.14 (*n* = 7)	7.0 (*n* = 6)	3.5 (*n* = 3)
	McDonald’s Total (*N* = 199)	61.3 (*n* = 122)	13.6 (*n* = 27)	44.2 (*n* = 88)	14.6 (*n* = 29)	8.5 (*n* = 17)	4.0 (*n* = 8)
KFC	High Income (*N* = 44)	84.1 (*n* = 37)	9.1 (*n* = 4)	2.3 (*n* = 1)	0 (*n* = 0)	0 (*n* = 0)	0 (*n* = 0)
	Upper-Middle-Income (*N* = 42)	69.1 (*n* = 29)*	0 (*n* = 0)	4.8 (*n* = 2)	7.1 (*n* = 3)	4.8 (*n* = 2)	0 (*n* = 0)
	Lower-Middle-Income (*N* = 32)	59.4 (*n* = 19)**	34.4 (*n* = 11)**	0 (*n* = 0)	9.4 (*n* = 3)	0 (*n* = 0)	9.4 (*n* = 3)
	KFC Total (*N* = 118)	72.0 (*n* = 85)	12.7 (*n* = 15)	2.5 (*n* = 3)	5.08 (*n* = 6)	1.7 (*n* = 2)	2.5 (*n* = 3)
Grand Total	(*N* = 406)	65.76 (*n* = 267)	15.52 (*n* = 63)	22.41 (*n* = 91)	10.10 (*n* = 41)	6.9 (*n* = 28)	7.9 (*n* = 32)

*p < .05 (comparison between HIC and UMIC)

**p < .05 (comparison between HIC and LMIC)

^a^Fisher’s Exact Test

**Table 2:** Descriptive summary of the nutritional quality of products advertised by food and beverage companies, ranked by country economic classification

Company	Country (*N* = total # of screenshots in income bracket)	NPI Scores (*N* = # of websites that had nutrition information)	% of Screenshots Showing Fresh Main Dishes	% of Screenshots Showing Fried Main Dishes	% of Screenshots Showing Cooked Main Dishes	% of Screenshots Showing Fresh Side Dishes	% of Screenshots Showing Fried Side Dishes	% of Screenshots Showing Cooked Side Dishes	% of Screenshots Showing Fresh Garnishes	% of Screenshots Showing Fried Garnishes	% of Screenshots Showing Cooked Garnishes
Coca-Cola	High Income (*N* = 17)	0 (*n* = 0)	0 (*n* = 0)	0 (*n* = 0)	5.8 (*n* = 1)	0 (*n* = 0)	0 (*n* = 0)	5.8 (*n* = 1)	5.8 (*n* = 1)	0 (*n* = 0)	0 (*n* = 0)
	Upper-Middle-Income (*N* = 24)	0 (*n* = 0)	0 (*n* = 0)	0 (*n* = 0)	0 (*n* = 0)	0 (*n* = 0)	0 (*n* = 0)	0 (*n* = 0)	4.1 (*n* = 1)	0 (*n* = 0)	0 (*n* = 0)
	Lower-Middle-Income (*N* = 48)	0 (*n* = 0)	0 (*n* = 0)	0 (*n* = 0)	0 (*n* = 0)	0 (*n* = 0)	0 (*n* = 0)	2 (*n* = 1)	0 (*n* = 0)	0 (*n* = 0)	0 (*n* = 0)
	Coca-Cola’s Total (*N* = 89)	0 (*n* = 0)	0 (*n* = 0)	0 (*n* = 0)	1.1 (*n* = 1)	0 (*n* = 0)	0 (*n* = 0)	2.5 (*n* = 2)	2.5 (*n* = 2)	0 (*n* = 0)	0 (*n* = 0)
McDonald’s	High Income (*N* = 27)	12.8 (*n* = 2)	14.8 (*n* = 4)	33.3 (*n* = 9)	48.1 (*n* = 13)	29.6 (*n* = 8)	33.3 (*n* = 9)	7.4 (*n* = 2)	37 (*n* = 10)	0 (*n* = 0)	0 (*n* = 0)
	Upper-Middle-Income (*N* = 86)	0 (*n* = 0)	2.3 (*n* = 2)*^a^	36.3 (*n* = 32)	43.1 (*n* = 38)	4.5 (*n* = 4)*^a^	25 (*n* = 22)	4.5 (*n* = 4)	10.2 (*n* = 9)*^a^	3.4 (*n* = 2)	2.3 (*n* = 2)
	Lower-Middle-Income (*N* = 86)	16.5 (*n* = 2)	2.3 (*n* = 2)**^a^	32.5 (*n* = 28)	40.7 (*n* = 35)	1.1 (*n* = 1)**^a^	10.5 (*n* = 9)**^a^	1.2 (*n* = 1)	1.1 (*n* = 11)**^a^	10.4 (*n* = 9)	1.1 (*n* = 1)
	McDonald’s Total (*N* = 199)	12.7 (*n* = 4)	4 (*n* = 8)	34.3 (*n* = 69)	42.7 (*n* = 86)	6.5 (*n* = 13)	26.4 (*n* = 53)	3.5 (*n* = 7)	10 (*n* = 20)	6 (*n* = 12)	1.5 (*n* = 3)
KFC	High Income (*N* = 44)	4.4 (*n* = 2)	0 (*n* = 0)	52.3 (*n* = 23)	9 (*n* = 4)	20.5 (*n* = 9)	6.8 (*n* = 3)	38.6 (*n* = 17)	9 (*n* = 4)	9 (*n* = 4)	0 (*n* = 0)
	Upper-Middle-Income (*N* = 42)	10 (*n* = 1)	0 (*n* = 0)	47.6 (*n* = 20)	28.5 (*n* = 12)*	9.5 (*n* = 4)	33.3 (*n* = 14)*	21.4 (*n* = 9)	7.1 (*n* = 3)	28.6 (*n* = 12)*	0 (*n* = 0)
	Lower-Middle-Income (*N* = 32)	0 (*n* = 0)	0 (*n* = 0)	62.5 (*n* = 20)	31.2 (*n* = 10)**	0 (*n* = 0)**^a^	15.6 (*n* = 5)	9.3 (*n* = 3)**^a^	15.6 (*n* = 5)	18.7 (*n* = 6)	0 (*n* = 0)
	KFC’s Total (*N* = 6)	7.2 (*n* = 3)	0 (*n* = 0)	53.4 (*n* = 63)	22 (*n* = 26)	11 (*n* = 13)	18.6 (*n* = 22)	18.6 (*n* = 26)	10 (*n* = 12)	18.6 (*n* = 22)	0 (*n* = 0)
Grand Total	(*N* = 406)	8.1 (*n* = 7)	2 (*n* = 8)	32.3 (*n* = 132)	27.7 (*n* = 113)	6.4 (*n* = 26)	18.4 (*n* = 75)	8.6 (*n* = 35)	8.3 (*n* = 34)	8.3 (*n* = 34)	1 (*n* = 3)

*p < .05 (comparison between HIC and UMIC)

**p < .05 (comparison between HIC and LMIC)

^a^Fisher’s Exact Test

### Comparisons between economic groupings across all three companies

High-income countries’ websites promoted a diet/healthy food (e.g. baked chicken sandwich) or beverage product on 25% (*N* = 22) of their pages, compared to 11·2% (*N* = 17) of the UMICs websites and 14·5% (*N* = 24) of the LMICs websites. Promotions were featured more often on webpages in HICs (12·5%) and UMICs (12·5%) than LMICs (6·6%). Across all three brands, links promoting a children’s section of the website appeared most frequently on UMICs’ websites. On average, links to a children’s section or promotional material directed at children (e.g. an image or text reference to children or cartoon characters) appeared on 4·6% (*N* = 4) of HICs’ webpages, compared to 38·2% (*N* = 58) of UMICs’ webpages and 17.7% (*N* = 29) in LMICs’ webpages. LMICs’ webpages were the most likely to reference a charity (15·7%, *N* = 26) compared to UMICs (2·6%, *N* = 4) and HICs (2·3%, *N* = 2). The proportion of webpages referencing a charity was significantly different between LMICs and UMICs (*p* < 0.001) as well as between LMICs and HICs (*p* = 0.001). Researchers also found some similarities in marketing techniques across economic groupings. More than 50% (*N* = 267) of all webpages showed any food or beverage product (i.e. diet or non-diet), and less than 10 % of all countries’ webpages referenced exercise or sports.

Fried main dishes were featured similarly across country economic groupings, while fresh main dishes (e.g. salads) were rarely featured on any website (*N* = 8). Furthermore, cooked main dishes were featured more frequently on UMICs websites. Fresh side dishes and garnishes were featured more frequently on HICs websites, while fried side dishes were featured more frequently on UMICs websites and fried garnishes were most frequently featured on LMIC’s websites (Table 2).

### Comparing different countries’ websites within the same parent company

An analysis of Coca-Cola’s marketing strategies showed that the ratio of SSBs to diet beverages differed between each economic country group. In the HICs, 88·2% (*N* = 15) of the webpages showed SSBs while 41·2% (*N* = 7) of the webpages promoted diet versions of beverages. Seventy-nine percent (*N* = 19) of the webpages in UMICs advertised sugar-sweetened beverages while 29·2% (*N* = 7) promoted a diet beverage. In LMICs, 54·2% (*N* = 26) of Coca-Cola’s webpages advertised sugar-sweetened beverages, while 14·6% (*N* = 7) promoted diet options. Taken together, the difference between how many times diet beverages versus SSBs appeared was narrower in HIC websites (where the split was 88% SSB versus 41% diet), whereas in UMIC and LMIC websites, the gap was wider (54% SSBs versus 14% diet) suggesting diet products are promoted less frequently to LMICs via these websites. The number of screenshots depicting diet alternatives was statistically significantly higher in HICs as compared to LMICs.

Within the McDonald’s company, fresh main dishes were advertised significantly more frequently on HIC websites (14·8%) than on UMIC and LMIC websites (2·3% each). Furthermore, fresh side dishes were marketed on 29·6% of the webpages in HICs, 4·5% in UMICs, and 1·1% in LMICs; these differences were also statistically significant. Diet products or healthy alternatives to standard fast food products were not offered at similar rates across country websites. Fifty-one percent of the LMICs’ webpages showed food or beverages, yet only 7% of these pages promoted diet products. Similarly, 67% of the UMICs’ webpages advertised food or beverages, and just 11.6% showed diet options. In comparison, 74% of the HICs webpages showed food or beverages while diet options were promoted on 40·7% of the webpages. The percentage of websites showing diet products or healthy alternatives was significantly different between HIC and UMIC as well as between HIC and LMIC. In addition to the variations between food marketing, 18·5% of the webpages in HICs referenced exercise or sports compared to 7.0% in UMICs, and 7.0% in LMICs.

Similar to Coca-Cola and McDonald’s, KFC promoted any food and beverages on a high percentage of its webpages. Although KFC’s webpages among all of the economic country groups did not advertise any fresh main dishes, 20·5% of HIC webpages promoted a fresh side dish, compared to 9·5% of UMIC webpages and 0% of LMIC pages. KFC webpages contained promotions on 7.1% of UMIC webpages, compared to 9.4% of LMIC webpages. No promotions were offered on HIC webpages.

## Discussion

In this study, we found that all three fast food and beverage companies promoted diet food or beverage products and fresh main dishes more frequently on their websites in HICs than in the UMICs and LMICs. However, fried main dishes and fried side dishes were displayed at similar rates across high-income countries. Promotions (e.g. free cell phone minutes) appeared more frequently on HICs and UMICs websites, and nutritional information was found on less than 40% of websites across all economic classifications. LMICs’ webpages were the most likely to reference a charity (15·7%, *N* = 26) compared to UMICs (2·6%, *N* = 4) and HICs (2·3%, *N* = 2). These findings suggest that companies promote fewer healthful products on their websites in lower-income populations while simultaneously highlighting their philanthropic activities to appeal to consumers in lower income countries. When comparing the practices of each company, Coca-Cola promoted diet beverages less frequently in lower income countries. The most striking contrast occurred with McDonald’s because half of the foods promoted on HIC websites were diet/healthy options, whereas just 6% of UMIC websites promoted healthy options on McDonald’s sites, and zero LMIC websites included diet/healthy options. Similarly, McDonald’s promoted more fresh main dishes on HIC websites compared to lower income country websites, and none of the web pages on LMIC websites showed diet products. A similar contrast appeared with KFC’s use of fresh side dishes on websites across different economic groupings. These differences in promotion of unhealthy food and SSBs are concerning because they occur in the context of the increasing burden of chronic diseases globally, with 77% of the world’s diabetic population residing in low- and middle-income countries [47]. In regards to physical activity messages, McDonald’s promoted exercise imagery more often on LMIC websites than their HIC websites. These messages, while important, need to be paired with healthy food messaging in part because research has shown that consumers perceive athlete-endorsed food as healthier than non-endorsed food [48]. Such messages create a “halo effect” in which consumers underestimate calories of food due to their marketing claims or visual appearance [49].

While no studies have compared the marketing techniques on food and beverage company websites across countries of varying economic status, other studies have examined the nutritional content of products promoted on websites. Previously published research on food websites have been mainly conducted in the US and have mostly focused on child-targeted content. Studies found that the majority of food and beverage products advertised on children’s websites are unhealthy [9–11]. Advergames websites (i.e. sites that offer online games branded with company logos) [7, 8] and cereal websites [12] also primarily promote unhealthy and sugary foods. In fact, Ustjanauskas et al. found that 84% of ads on popular children’s websites show products high in fat, sugar, and/or sodium [9]. Lingas et al. found that only five out of 77 food and beverage products advertised on popular children’s websites met the Institute of Medicine guidelines for foods to promote to youth [10]. In terms of methods, food advertising on websites use branded engagement techniques and themes of identity formation to specifically capture the attention of children [50].

Our findings were similar to some previously published research on website-based food marketing in that the majority of products promoted were energy-dense, nutrient-poor products, and companies used a variety of promotional techniques. However, our results show fewer child-targeted marketing practices than those studies, likely because a number of those studies focused specifically on child-targeted advergames. The inclusion of corporate websites that are owned by the same parent company but span a variety of countries from different economic groups is a strength of this study because it enabled us to compare the different promotional techniques across countries and within each company. Indeed, this is the first study to our knowledge to identify some differences in website-based marketing techniques across higher and lower income countries within the same parent companies. Still, the current study is limited by the possibility that researchers missed some marketing techniques or cooking methods shown on the webpages. Furthermore, the screenshots were taken of only six countries’ websites, limiting our ability to make broader conclusions about these companies’ international food and beverage marketing techniques. Lastly, because we did not experimentally assess the influence of advertised food and beverage products on consumer or health behaviors, the relationship between purchase behaviors or consumption behaviors with website content cannot be determined from these data.

Future research should examine international digital food marketing by broadening the number of countries and companies examined and by comparing the marketing techniques and nutritional quality of products promoted on food companies’ international social media accounts. Similar to the way food companies maintain official websites for different country, many companies have social media accounts on Twitter and Facebook that are specific to different countries. It is unclear how many youth engage with food companies’ social media accounts, suggesting a need for thorough surveillance data that could inform the extent to which companies should participate in uniform guidelines about marketing to youth through social media [51]. For example, self-regulatory efforts include the International Chamber of Commerce Guidelines on Advertising, Federation of European Direct Marketing Code on E-Commerce and Interactive Marketing, and the European Group on Television Advertising (EGTA) Guidelines for Commercial Communications on New Interactive Services are all designed to eliminate the risk of harm or exploitation of youth online and could be useful models for implementing uniform guidelines. Regulating digital marketing is complex because of cross-border marketing, [51] but critical for reducing youth exposure to unhealthy food and beverage ads.

One other notable finding that was not part of our aims involves the lack of nutritional information available on McDonald’s Philippines website despite being present elsewhere. Additionally, McDonald’s in India is divided into two separate sites; one for the historically wealthier south and one for the rural and historically poorer north [52]. There were several notable discrepancies in the marketing techniques used on the two McDonald’s India websites: while 100% of northern India pages contained promotional material aimed at children, only 13% of southern India pages contained such material. In addition, 50% of southern India pages showed a fresh main dish while only 13% of northern India pages showed a fresh main dish.

## Conclusions

This study finds that some fast food and beverage companies are marketing healthier products in wealthier countries and showcasing their philanthropic activities in lower income countries. Overall, economic globalization has enabled food and beverage marketers to introduce a variety of reasonably priced, [53] culturally resonant products and market them to responsive audiences, but these companies should also take responsibility for the influence their products can have on the populations they target. These findings can be used to guide the development of policies to address the growth of fast food and beverage marketing in developing and emerging markets in LMICs and UMICs. Policymakers could consider prohibiting the use of certain marketing techniques in their countries or advocate for the promotion and sale of healthier food and beverage products.

## Additional file

Additional file 1: Figure S1. Overview of Qualitative Content Analysis Coding Process. Flow chart explaining the coding process. (PDF 123 kb)

## Abbreviations

HIC: High income country
KFC: Kentucky Fried Chicken
LMIC: Lower-middle-income country
NPI: Nutrient Profile Index
NPM: Nutrient Profile Model
OECD: Organization for Economic Co-Operation and Development
SSB: Sugar sweetened beverage
UMIC: Upper-middle-income country
