# Supplementary figures and images for: Comparison of online marketing techniques on food and beverage companies’ websites in six countries
Source: Global Health. 2017 Oct 26;13:79. doi: 10.1186/s12992-017-0303-z (PMC5658924; doi:10.1186/s12992-017-0303-z)

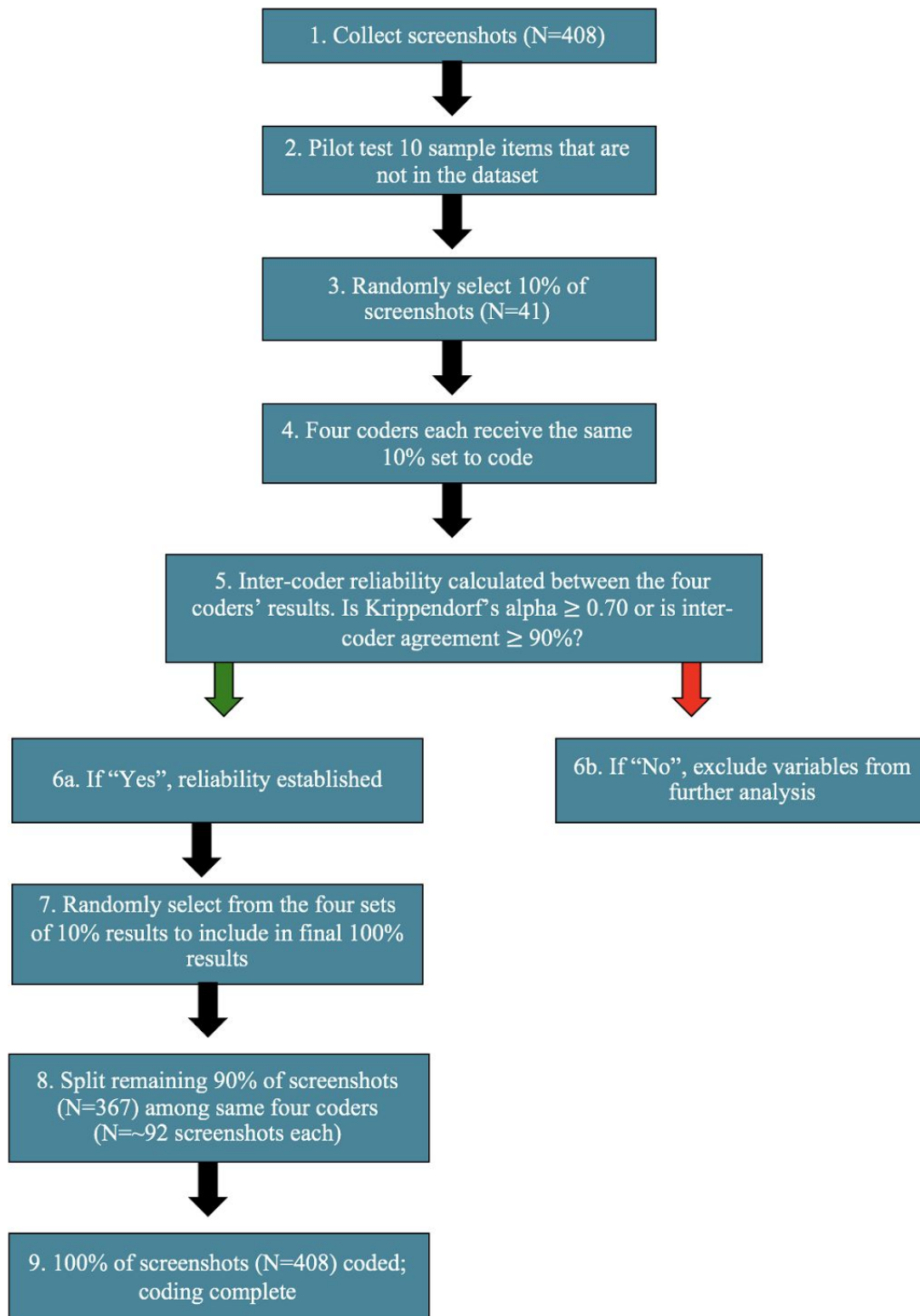

Supplement: Additional file 1: Figure S1. — Overview of Qualitative Content Analysis Coding Process. Flow chart explaining the coding process. (PDF 123 kb) [file 12992_2017_303_MOESM1_ESM.pdf]
